# Unravelling genetic variants of a swedish family with high risk of prostate cancer

**DOI:** 10.1186/s13053-022-00234-0

**Published:** 2022-07-23

**Authors:** Serena Barilla, Annika Lindblom, Hafdis T. Helgadottir

**Affiliations:** 1 Department of Molecular Medicine and Surgery, Karolinska Institute, Solna, Stockholm Sweden; 2 Department of Clinical Genetics, Karolinska University Hospital, Solna, Stockholm Sweden

**Keywords:** Prostate cancer, Hereditary, Germline, Whole-genome sequencing

## Abstract

**Background:**

Prostate cancer is the most prevalent cancer in men worldwide. It is a polygenic disease with a substantial proportion of heritability. Identification of novel candidate biomarkers is crucial for clinical cancer prevention and the development of therapeutic strategies. Here, we describe the analysis of rare and common genetic variants that can predispose to the development of prostate cancer.

**Methods:**

Whole-genome sequencing was performed on germline DNA of five Swedish siblings which were diagnosed with prostate cancer. The high-risk variants were identified setting the minor allele frequency < 0.01, CADD > 10 and if tested in PRACTICAL, OR > 1.5, while the low-risk variants were identified minor allele frequency > 0.01, CADD > 10 and if tested in PRACTICAL, OR > 1.1.

**Results:**

We identified 38 candidate high-risk gene variants and 332 candidate low-risk gene variants, where 2 and 14 variants were in coding regions, respectively, that were shared by the brothers with prostate cancer.

**Conclusions:**

This study expanded the knowledge of potential risk factor candidates involved in hereditary and familial prostate cancer. Our findings can be beneficial when applying targeted screening in families with a high risk of developing the disease.

**Supplementary Information:**

The online version contains supplementary material available at 10.1186/s13053-022-00234-0.

## Background

Prostate cancer (PrCa) is the second most frequent cancer in men worldwide and the most common diagnosed cancer among men in the United States and most of the Western world [1]. PrCa is a heterogeneous disease that can be associated with several risk factors including age, race, familial history of PrCa, diet or environmental factors [2]. Epidemiological and twin studies had supported the pivotal role of genetic predisposition in the development of PrCa. A positive familial history of PrCa has been associated with more than a two-fold increased risk of the disease [3]. Furthermore, having a brother with PrCa represents a greater risk to be affected by the disease than being a son of a PrCa patient [4]. In addition, Scandinavia twins studies have demonstrated that the 42% of PrCa risk was due to heritable factors and the probability of having PrCa was 21% for a man with a monozygotic twin affected and 6% for a man with a dizygotic twin affected [5] with 3.7 year of difference between the diagnose of the first and second monozygotic twin [6].

For these reasons, germline mutations are becoming the focus of increased numbers of research to look for inherited variations that are suitable for potential prognosis and treatment. Although the androgen receptor (AR) has a pivotal role in PrCa, mutations in the AR gene account for a small fraction of all case [7], underling that different genetic variations may contribute to the formation of prostate tumors. Evidence has shown that the genetic contribution to inherited PrCa is constituted by a mixture of rare gene variants with high to moderate penetrance and common variants with low penetrance. Linkage studies have identified 8q24 as a significant PrCa risk region [8] and the missense G84E variant in the *HOXB13* [9] gene is highly associated with an increased risk of PrCa. It has been shown that variants in DNA repair genes, such as the *BRCA1/2*, *ATM*, *CHEK2* and *NBN* genes, are associated with an increased risk of developing PrCa, in particular in men with advanced/metastatic PrCa [10]. Retrospective and association studies have shown that carriers of pathogenic *BRCA2* variants are associated with two- to six-times higher relative risks of PrCa while carriers of pathogenic *BRCA1* variants are associated with a moderate risk [11, 12]. Moreover, the knowledge of a patient’s germline status has become of emerging importance, in predicting the response to targeted treatments, especially in advanced or metastatic conditions [13].

The polygenic nature of PrCa susceptibility has brought the attention into genome-wide association study (GWAS) and to the formation of large collaborative international consortia that includes thousands of cases and controls to detect PrCa risk variants. Since the first GWAS in 2006 [14], around 170 variants have been identified to be risk factors for PrCa susceptibility. However, they can only explain approximately 38% of the familial relative risk of PrCa [15] and additional evidence is required to include them in routine genetic testing.

Here, we present the results of five brothers with PrCa and previously shown not to be carrying any of the high-risk variants so far identified in the literature. Through whole-genome sequencing on their germline DNA we identified novel high- and low-risk genetic variants that could contribute to the PrCa risk observed in their family.

## Materials and methods

### Study population

The individuals in this study were five brothers from a Swedish family that had undergone genetic counselling at the Department of Clinical Genetics, Karolinska University Hospital Solna, Sweden. The five brothers were diagnosed with PrCa between the age of 64–80, and they had also a brother diagnosed with a neuroendocrine pancreatic tumor (NET) at the age of 79 years, and a seventh brother who was healthy (at the age of 61 years).

Additional family history of PrCa includes their father and paternal uncle were diagnosed with PrCa. No other cancer was known in the family on the mother´s or father´s side. All seven brothers gave written informed consent to participate to the study and DNA was isolated from peripheral blood.

### Whole-genome sequencing of prostate cancer family samples

DNA was quantified using a Qubit Fluorometer (Life Technologies, USA) and converted to sequencing libraries using a PCR-free paired-end protocol (Illumina TruSeq DNA PCR-free for > 1000 ng input). Sequencing was done using the Illumina NovaSeq 6000 platforms aiming at 30x median coverage and performed at Clinical Genomics Stockholm, SciLifeLab.

### Bioinformatics workflow

Sequencing reads were aligned to the reference genome GRCh37 using BWA [16] and Picard (http://picard.sourceforge.net) was used to mark PCR-duplicated reads. Variants were called using GATK best practice procedure as implemented at the Broad Institute (www.broadinstitute.org/gatk). Variant annotation was done using Ensembl Variant Effect Predictor (VEP) [17], including RefSeq gene annotation and dbSNP153. Max minor allele frequency (MMAF) was obtained from Genome Aggregation Database [18] (gnomAD), or from the SweGen database [19] when missing from gnomAD. To predict pathogenic effects of the variants, the *in silico* prediction database CADD (Combined Annotation Dependent Depletion) [20] was used, while to predict the associated risk factor for PrCa the odds ratio (OR) and *P* values from the PRACTICAL project [21] was used. Variants in the autosomal and sex chromosomes were analysed. Variants located within segmental duplication regions obtained from the UCSC genome browser [22] were excluded.

### Known hereditary cancer genes

Variants in 154 genes belonging to the Comprehensive Hereditary Cancer Panel (https://blueprintgenetics.com/) were investigated in the brothers to identify pathogenic variants. That includes the 10 genes (*ATM, BRCA1,BRCA2, PALB2, CHEK2, HOXB13, MLH1, MSH2, MSH6, PMS2*) recommended for genetic testing by the National Comprehensive Cancer Network guidelines for PrCa [23].

### High- and low-risk PrCa predisposing variants

Variants detected in all siblings with PrCa were divided in two groups: rare high-risk variants and common low-risk variants. Rare high-risk variants were considered variants with MMAF (gnomAD/SWEGEN) < 0.01, CADD > 10 and if tested in PRACTICAL [21], OR > 1.5. Common low-risk variants were considered variants with MMAF (gnomAD) > 0.01, CADD > 10 and if tested in PRACTICAL [21], OR > 1.1 and *P-* values < 0.05. For both our categories risk factors were also considered the variants shared by all brothers with PrCa and not imputed in the custom high-density genotyping array, the OncoArray, from the PRACTICAL project [21]. Further, for all variants a CADD score greater of equal 10 indicates that these are predicted to be the 10% most deleterious substitutions that you can do to the human genome, while a score of greater or equal 20 indicates the 1% most deleterious.

### Enrichment analysis

The annotated variants were imported in R and Bioconductor, the disgenet2r package [24] was used for the gene-neoplastic disease association analysis and neoplastic process enrichment analysis, while the clusterProfiler package [25] was used for or the gene ontology enrichment analysis.

## Results

### Identification of 38 novel candidate high-risk variants in inherited prostate cancer

To identify the PrCa predisposing genes we analysed the whole-genome sequence from five brothers which were diagnosed with PrCa. To test if they inherited genetic susceptibility to cancer we first checked if they had pathogenic variants in genes included in the comprehensive hereditary cancer panel (https://blueprintgenetics.com/tests/panels/hereditary-cancer/comprehensive-hereditarycancer-panel/#panel_content-headin) . We found 4 variants carried by all brothers but none of them had any known pathogenic consequences according to ClinVar (Table 1).

**Table 1:** Variants in coding regions of the genes included in the Comprehensive Hereditary Cancer carried by all siblings

Gene	Location^a^	Ref/Alt	SNP-id	HGVS transcript	Clinical significance
*MLH1*	chr3:37053568	A/G	rs1799977	NM_001167617.2:c.361 A > G	benign
*EZH2*	chr7:148525904	C/G	rs2302427	NM_001203248.1:c.526G > C	benign
*BRCA2*	chr13:32906729	A/C	rs144848	NM_000059.3:c.1114 A > C	benign
*ERCC2*	chr19:45854919	T/G	rs13181	NM_000400.3:c.2251 A > C	benign

^a^Genomic coordinates were based on GRCh37

Next, we searched for genetic variants that were shared by all sibling and we found 69,548 shared variants, of which 68,846 were in non-coding regions and 702 were in coding transcripts. Since the genetic contribution to inherited PrCa includes a variety of rare or common variants, we started our analysis of the shared variants by looking for high-risk candidates. First, we selected variants with MMAF < 0.01 and CADD > 10, to be defined as the most likely high-risk variants. We excluded variants with low risk (OR < 1.5) in the PRACTICAL study [21]. We identified 38 variants in 35 genes suggested to be associated with high PrCa risk. Most variants were intronic variants; however, two variants, rs150518260 and rs139884486, were found to be located within the coding regions of the *SGCB* and the *IRF4* genes, respectively and they were also the most deleterious substitution (CADD > 20) that we found within our high-risk variant (Table 2). Furthermore, we found that eight of the suggested genes (*NFIB, FBXW7, PPARA, ETV4, NCOA2, CYP7B1, OPCML, IRF4*) have been demonstrated to be involved in neoplastic processes (Supplementary Fig. 1 A) and four of them, the *ETV4*, *NCOA2*, *CYP7B1* and *PPARA* genes, have been shown to be associated with malignant neoplasms of the prostate (Supplementary Fig. 1B and Supplementary Table 1). None of the siblings carried the rare high-risk missense variant (*G84E*) on the *HOXB13* gene but they were carrying one upstream variant in the non-coding region of the *HOXB3* gene (Table 2).

**Table 2:** Rare and deleterious variants with MMAF < 0.01, CADD > 10, ODD Ratio > 1.5 shared by the five brothers with prostate cancer

Gene	Location^a^	Ref/Alt	SNP-id	HGVS transcript	MMAF^b^	MAF^c^	CADD^d^	Position	Consequence
*USH2A*	chr1:216090029	T/C	Na	NM_206933.2: c.7301-15782 A > G	4.53E-04	0	13.45	Transcript	Intronic
*ESRRG*	chr1:217149814	T/C	Na	NM_001350122.1: c.-284-36799 A > G	9.07E-04	0.0025	14.77	Transcript	Intronic
*RAB3GAP2*	chr1:220332126	C/A	Na	NM_012414.3: c.3261 + 602G > T	0	0	10.16	Transcript	Intronic
*OBSCN*	chr1:228395681	T/G	Na	NR_073154.1: n.709-1330 A > C	0.007435	0.004004	11.34	Transcript	Upstream
*UBE2E1*	chr3:23847362	A/T	Na	NR_046652.1: n.87 + 948T > A	0	0	15.69	Intergenic	Upstream
*GBE1*	chr3:81534434	C/A	rs139860257	NC_000003.11: g.81,534,434 C > A	0.001879	0.0075	17.48	Transcript	Downstream
*DLG1*	chr3:196990566	G/C	rs111626925	NM_001204386.1: c.318 + 18,984 C > G	1.15E-04	0	11.97	Transcript	Intronic
*PPARGC1A*	chr4:23981292	C/CG	Na	NM_001330751.1: c.70-94739dup	0.001881	0.002	16.87	Transcript	Intronic
*SGCB*	chr4:52895932	G/A	rs150518260	NM_000232.4: c.341 C > T	2.6E-04	5.00E-04	28	Coding Transcript	Non-synonymous
*LRBA*	chr4:151631987	C/T	Na	NM_001199282.2: c.5754 + 24423G > A	0.001621	5.00E-04	12.08	Transcript	Intronic
*FBXW7*	chr4:153359554	G/A	Na	NM_001257069. 1:c.-119-25873 C > T	0.001179	5.00E-04	10.92	Transcript	Intronic
*TLL1*	chr4:166827816	A/T	rs72695713	NM_001204760.1: c.169 + 32,591 A > T	0.005513	0.0075	11.69	Transcript	Intronic
*NEK1*	chr4:170464385	T/C	Na	NM_001199397.1: c.1563-5323 A > G	0.001750	0.001	11.43	Transcript	Intronic
*IRF4*	chr6:393175	G/A	rs139884486	NM_001195286.1: c.23G > A	3.9E-04	0.001	23.1	Coding Transcript	Non-synonymous
*CYP7B1*	chr8:65518926	G/T	rs185336165	NM_001324112.1: c.1058-1512 C > A	0.003953	0.009	12.35	Transcript	Intronic
*CSPP1*	chr8:68106087	T/G	Na	NM_001291339.1: c.2419 + 250T > G	0.001231	5.00E-04	10.44	Transcript	Intronic
*NCOA2*	chr8:71094518	A/G	rs182681059	NM_006540.3: c.260-7424T > C	6.48E-05	0	15.78	Transcript	Intronic
*KCNK9*	chr8:140695084	G/C	Na	NM_001282534.1: c.283 + 19,869 C > G	0	0	10.25	Transcript	Intronic
*NFIB*	chr9:14190548	G/A	Na	NM_001190738.1: c.641-10769 C > T	0	0	17.98	Transcript	Intronic
*NFIB*	chr9:14236935	C/T	Na	NM_001190738.1: c.641-57156G > A	0	0	19.88	Transcript	Intronic
*NFIB*	chr9:14242536	G/A	Na	NM_001190737.1: c.563-62757 C > T	0.004408	0.002	18.8	Transcript	Intronic
*NFIB*	chr9:14302801	G/A	Na	NM_001190738.1: c.640 + 4187 C > T	1.29E-04	0.002	17.56	Transcript	Intronic
*HPSE2*	chr10:100787305	A/G	Na	NM_001166244.1: c.610 + 116690T > C	0.006031	0.0035	11.3	Transcript	Intronic
*OPCML*	chr11:132891613	G/C	rs5029236	NM_001012393.1: c.62-78708 C > G	6.04E-04	0	18.19	Transcript	Intronic
*FGD4*	chr12:32798780	T/G	Na	NM_001304480.1: c.*5313T > G	0	0	11.59	Transcript	3prime-UTR
*ANO6*	chr12:45663112	T/C	Na	NM_001204803.1: c.71-1133T > C	0	0	17.42	Transcript	Intronic
*FREM2*	chr13:39384076	T/C	Na	NM_207361.4: c.6019 + 25131T > C	0	0	10.96	Transcript	Intronic
*DIAPH3*	chr13:60308696	C/A	rs118098467	NM_001042517.1: c.3319 + 39627G > T	0.001880	0.0045	17.56	Transcript	Intronic
*DNAJC3*	chr13:96328151	A/G	rs17884852	NR_132117.1: n.1029T > C	0.002852	0.003	10.43	Transcript	Upstream
*CLYBL*	chr13:100285053	A/G	Na	NM_206808.3: c.62 + 26,042 A > G	0	0	10.62	Transcript	Intronic
*ETV4*	chr17:41632099	G/C	Na	NC_000017.10: g.41632099G > C	0	0	13.68	Transcript	Intronic
*GOSR2*	chr17:45028738	C/G	rs190194114	NM_001321133.1: c.583 + 12,668 C > G	0.002205	0.004	15.59	Transcript	Intronic
*HOXB3*	chr17:46669864	A/AGGGAGGGGGCACTGGGT	Na	NM_002147.3: c.563 − 63_563-47dup	0.001217	0	16.21	Transcript	Upstream
*CA4*	chr17:58228301	T/G	Na	NM_000717.3: c.58 + 848T > G	0.001562	0	10.22	Transcript	Intronic
*SOCS6*	chr18:67956480	C/T	Na	NM_004232.3: c.-127 + 154 C > T	0.002323	0.0035	12.83	Transcript	Intronic
*STK35*	chr20:2129173	G/T	Na	NM_080836.3: c.*4781G > T	0	0	14.3	Transcript	3prime-UTR
*KCNJ6*	chr21:39075146	A/G	Na	NM_002240.3: c.946 + 11368T > C	0.003114	0.005	10.96	Transcript	Intronic
*PPARA*	chr22:46601451	A/T	rs4823477	NM_001001928.3: c.208 + 6963 A > T	0	0	11.53	Transcript	Intronic

^a^Genomic coordinates were based on GRCh37, ^b^ Max minor allele frequency (MMAF) obtained from Genome Aggregation Database (gnomAD), ^c^ Minor allele frequency (MAF) obtained from SWEGEN database, ^d^ Combined Annotation Dependent Depletion (CADD) *in silico* prediction of variants pathogenic effect

### Identification of novel candidate low-risk variants in inherited prostate cancer

Since the five siblings affected by PrCa were sharing a high number of variants, we decided, to study also low-risk PrCa candidates. All variants shared by the five affected brothers with MMAF > 0.01, CADD > 10 and OR > 1.1 for those being tested in the PRACTICAL study, were selected for further analysis.In total, 332 variants in 225 genes (Supplementary Table 2) were suggested as potential low-risk candidates in familial PrCa. We found that most of the variants, 227, were in the non-coding transcript region, while 88 variants were in the intragenic region and 17 in coding regions (Table 3). Interestingly, we found that the selected genes have been described in association with several neoplastic diseases with PrCa among the top enriched neoplastic process (Supplementary Fig. 2 A). Gene ontology analysis showed that these genes were enriched for pathways involved in early development but also for pathways related to cell division, DNA replication and apoptotic signaling (Supplementary Fig. 2B). Fourteen variants with potential pathogenic effect (non-synonymous, frameshift, in-frame deletion) were seen in 12 genes (Table 4), where four of the genes (*KRT18*, *TET2*, *IL32*, *SMPD1*) have already been shown to be associated with PrCa (Supplementary Fig. 2 C). Moreover, nine of this fourteen variants located in *TET2*, *NOP16*, *PRIM2*, *CACNA1B*, *PSMD13*, *SMD13*, *KRT18*, *MEF18* and *KRT10* genes belong to the 1% most deleterious substitution in the human genome with a CADD score > 20 (Table 4). Among the low-risk variants all siblings were carrying two upstream variants in the non-coding regions of the *HOXB3* gene and one downstream variant in the intragenic region of the *HOXB6* genes (Supplementary Table 2). In addition, we found that two variants in the *KRT18* gene (chr12:53343318 C > T and chr12:53343325T > A) were located on the same allele.

**Table 3:** Overview of low-risk variant consequences with MMAF > 0.01, CADD > 10 and ODD Ratio > 1.1 shared by the brothers with prostate cancer

Position	Total number	Variant consequence	Number
Non-coding transcript	227	3-prime-UTR	8
		5-prime-UTR	11
		Intronic	208
Intragenic	88	Upstream	49
		Downstream	36
		Intragenic	3
Coding transcript	17	Non-synonymous	7
		Synonymous	3
		Inframe-deletion	4
		Frameshift	3

**Table 4:** Low risk variants with MMAF > 0.01, CADD > 10, ODD Ratio > 1.1 in coding transcript shared by all brothers with prostate cancer

Gene	Location^a^	Ref/Alt	SNP-id	HGVS transcript	MMAF^b^	CADD^c^	Position	Consequence
*TET2*	chr4:106196829	T/G	rs34402524	NM_001127208.2: c.5162T > G	0.1234	22	Coding Transcript	Non-synonymous
*NOP16*	chr5:175811094	C/CAT	rs56989856	NM_001291305.2: c.546_547insAT	0.1973	22.1	Coding Transcript	Frameshift
*PRIM2*	chr6:57398226	T/G	rs77436138	NC_000006.11: g.57437519T > C	0.2653	21.8	Coding Transcript	Non-synonymous
*MAFA*	chr8:144511953	ATGGTGG /A	rs141816879	NM_201589.3: c.618_623del	0.0777	17.43	Coding Transcript	Inframe deletion
*CACNA1B*	chr9:140777299	G/T	rs71238527	NM_000718.3: c.494G > T	0.1791	24.7	Coding Transcript	Non-synonymous
*PSMD13*	chr11:247329	C/T	rs28927679	NM_002817.3: c.449 C > T	0.0106	23.4	Coding Transcript	Non-synonymous
*SMPD1*	chr11:6412853	G/GC	Na	NM_001318087.1: c.564dup	0.0170	20.9	Coding Transcript	Frameshift
*KRT18*	chr12:53342968	C/T	rs76301931	NM_000224.2: c.11 C > T	0.1759	16.1	Coding Transcript	Non-synonymous
*KRT18*	chr12:53343318	C/T	Na	NM_000224.2: c.361 C > T	0.1235	23.3	Coding Transcript	Non-synonymous
*KRT18*	chr12:53343325	T/A	Na	NM_000224.2: c.368T > A	0.0658	23.8	Coding Transcript	Non-synonymous
*MEF2A*	chr15:100252709	CCAGCAG/T	rs72276751	NM_001365203.1: c.1286_1291del	0.3553	22.2	Coding Transcript	Inframe deletion
*IL32*	chr16:3119297	C/CG	rs71818662	NM_004221.4: c.514dup	0.3126	18.43	Coding Transcript	Frameshift
*KRT10*	chr17:38975307	TGCCGCCGTGGCC/T	Na	NM_000421.3: c.1468_1479del	0.3077	20.2	Coding Transcript	Inframe deletion
*CD3EAP*	chr19:45912489	CAAG/C	rs374686338	NM_001983.3: c.*441_*443del	0.2206	13.39	Coding Transcript	Inframe deletion

^a^ Genomic coordinates were based on GRCh37, ^b^ Max minor allele frequency (MMAF) obtained from Genome Aggregation Database (gnomAD), ^c^ Combined Annotation Dependent Depletion (CADD) *in silico* prediction of variants pathogenic effect

## Discussion

To identify potential variant contribution to hereditary PrCa, we sequenced the whole genome of siblings in a family with a seemingly high risk of PrCa. As expected less than 1% of the shared variants were located within protein-coding regions of the genome. Although the stability, the structure and biochemical function of the proteins are important factors implicated in the development of PrCa, several other mechanisms that regulate the transcriptional and translational level of the protein may have a pivotal role in driving tumorigenesis.

We initially screened for rare variants that could potentially represent high-risk factors in the development of PrCa. As a second approach we searched for variants considered of low-risk impact in the development of the disease. Two candidate high-risk missense variants were found in the *SGCB* and the *IRF4* genes. The *SGCB* gene has not previously been linked to any neoplastic disease, mutations in this gene have been associated with the development of limb-girdle muscular dystrophy type 2E [26]. Instead, IRF4, an important regulator of the immune response, has been shown to be associated with many lymphoid malignancies with evidence pointing to a pivotal role in multiple myeloma [27]. Since inflammation is a risk factor for prostate carcinogenesis it could be possible that variations of the *IFR4* gene may contribute to increased risk of PrCa. Moreover, we found that four of our high-risk variants were situated in genes earlier demonstrated to be involved in the initiation and progression of PrCa. It has been shown that ETV4 may be involved in the initial events of PrCa development when it is fused with the *TMPRSS22* locus [28]. The expression of NCOA2 has been demonstrated to promote PrCa metastasis and its inhibition has therapeutic potential [29]. The expression of CYP7B1 has been shown to be upregulated in prostate cancer [30] and it is also been demonstrated that a single polymorphism in the promoter of this gene has an effect on its expression [31]. Furthermore, we observed a variant in the *PPARA* gene, which is overexpressed in advanced PrCa [32]. Variants in this gene have been associated with pesticide exposure and increased risk of PrCa [33] underling the importance of gene-environment interaction as a contributing factor to increased risk of PrCa.

In our analysis, we did not find pathogenic variants in any of the genes recommended for genetic testing by the National Comprehensive Cancer Network guidelines for PrCa [23], however in our high-risk group we found that they were carrying one novel variant in the upstream non coding region of the *HOXB3* gene. Further, in our low-risk category we found that they were carrying two novel variants in the *HOXB3* gene and one novel variant in the *HOXB6* gene but none of them was carrying the well know high-risk missense variant (G84E) in *HOXB13* gene [9]. Although none of the sibling was carrying the rare *HOXB13* mutation (G84E) we could suggest, based on our results, that also *HOXB3* and *HOXB6* genes may be PrCa susceptibility genes and germline mutation on these genes can play a role in predisposing the disease. The variation in the *HOXB3* and *HOXB6* genes that we found where located in upstream and downstream intragenic region and misregulation of the expression of these genes may lead to changes of the downstream gene expression and signaling pathways that play fundamental roles in the development of PrCa.

We identified several candidates as low-risk variants, as well as two missense variants, rs34402524 and rs28927679 in the *TET2* and *PSMD13* genes, respectively, previously shown to be associated with PrCa low-risk (OR of 1.1) [21]. It is well known that aside genetic factors also epigenetic factors can contribute to the initiation and progression of PrCa [34]. TET2 is an enzyme involved in DNA demethylation, and it has been shown to be an important player during tumorigenesis [35]. TET2 has been shown to be able to bind to the androgen receptor and modulate its signalling pathway. Downregulation of TET2 drives the PrCa proliferation and is strongly associated with reduced patient survival suggesting that the expression levels of this protein can be used as a biomarker for PrCa progression [36]. DNA methylation studies have demonstrated that DNA methylation patter is often altered in cancer compared to normal tissue [37]. This underline that a better understanding of the role DNA methylases including the TET family could be a promising therapeutic target to classify and predict PrCa clinical outcomes more accurately than clinical parameters alone. Interestingly, we found three variants in the *KRT18* gene, previously linked to PrCa. KRT18 is a well-known epithelial marker in diagnostic histopathology [38] and its downregulation is associated with prostate cancer aggressiveness [39].

This study has several limitations that need to be considered. The cohort of patients is limited to a single high-risk family containing five brothers with PrCa. The study does not include other affected or unaffected in the family. Strict criteria to identify high- and low-risk variants using population frequency excludes more common variants that could contribute to increased risk of PrCa. Furthermore, *in silico* prediction programs may be inaccurate resulting in exclusion of variants of high impact. Our variants are not validated with a secondary method or did we identify the variants in additional high-risk families. Moreover, our study does not include analysis of epigenetic variation that could contribute to the increased risk of PrCa of this particular family.

## Conclusions

PrCa is a complex disease which risk can be influenced by several genes and pathways. The identification of risk genes is crucial for genetic counselling of PrCa families and to apply the proper therapeutic strategies. The effect of a single genetic variant on the relative risk is probably low. Our study provided additional support that cumulative variants with low- or moderate effect in the germline of an individual contribute to the risk of PrCa. Further studies are needed to estimate the contribution of these variants and genes to PrCa.

## Supplementary Information

**Additional file 1.**

## Data Availability

Access to the data is controlled. Variants that fulfilled our selection criteria can be found in the tables and supplementary tables. However, Swedish laws and regulations prohibit the release of individual and personally identifying data. Therefore, the whole data cannot be made publicly available. The data that support the findings of this study are available from the corresponding author upon reasonable request.

## Abbreviations

PrCa: prostate cancer
AR: androgen receptor
GWAS: genome-wide association study
*HOXB3/6/16*: Homeobox B3/6/16
*BRCA1/2*: Breast cancer type1/2
*ATM*: ATM Serine/Threonine Kinase
*CHEK2*: Checkpoint Kinase 2
*NBN*: Nibrin
*SGCB*: Sarcoglycan Beta
*IRF4*: Interferon Regulatory Factor 4
*NFIB*: Nuclear Factor I B
*PPARA*: Peroxisome Proliferator Activated Receptor Alpha
*ETV4*: ETS Variant Transcription Factor 4
*NCOA2*: Nuclear Receptor Coactivator 2
*ESRRG*: Estrogen Related Receptor Gamma
*OPCML*: Opioid Binding Protein/Cell Adhesion Molecule Like
*KRT18*: Keratin 18
*TET2*: Tet Methylcytosine Dioxygenase 2
*IL32*: Interleukin 32
*SMPD1*: Sphingomyelin Phosphodiesterase 1
