# Supplementary material for: Unravelling genetic variants of a swedish family with high risk of prostate cancer
Source: Hered Cancer Clin Pract. 2022 Jul 23;20:28. doi: 10.1186/s13053-022-00234-0 (PMC9308349; doi:10.1186/s13053-022-00234-0)
Supplement: Supplementary file 1 — Additional file 1. [file 13053_2022_234_MOESM1_ESM.zip › Supplementary Figures&TablesR2.pdf]

Supplementary Figure 1

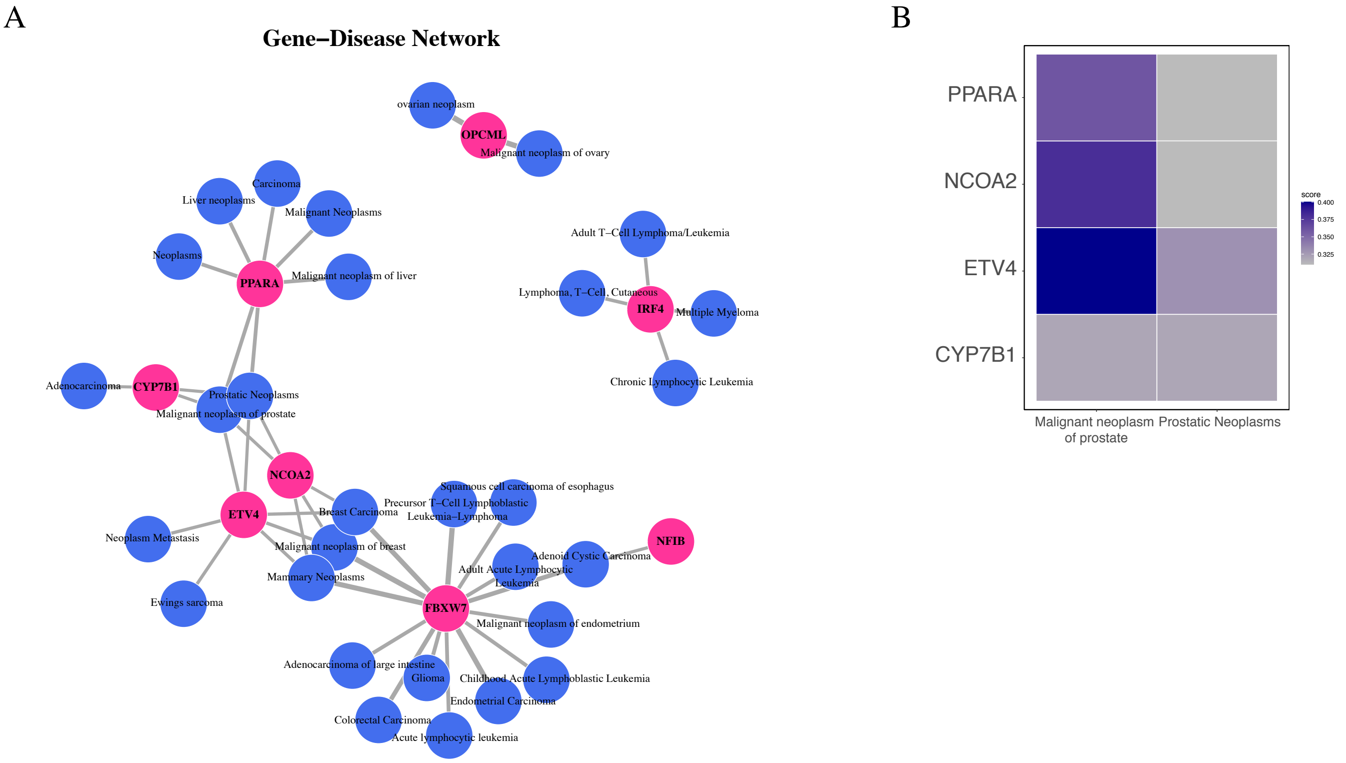

Supplementary Figure 1: Rare and deleterious variants analysis. (**A**) Gene-disease network for neoplastic process for the list of genes in high-risk variants. The edges represent the diseases, the pink nodes are the genes, and the width of the edges is proportional to the score of the association. (**B**) Gene-disease association heatmap for the list of genes in high-risk variants. Score based on the gene disease association performed with disgenet2r package.

Supplementary Figure 2

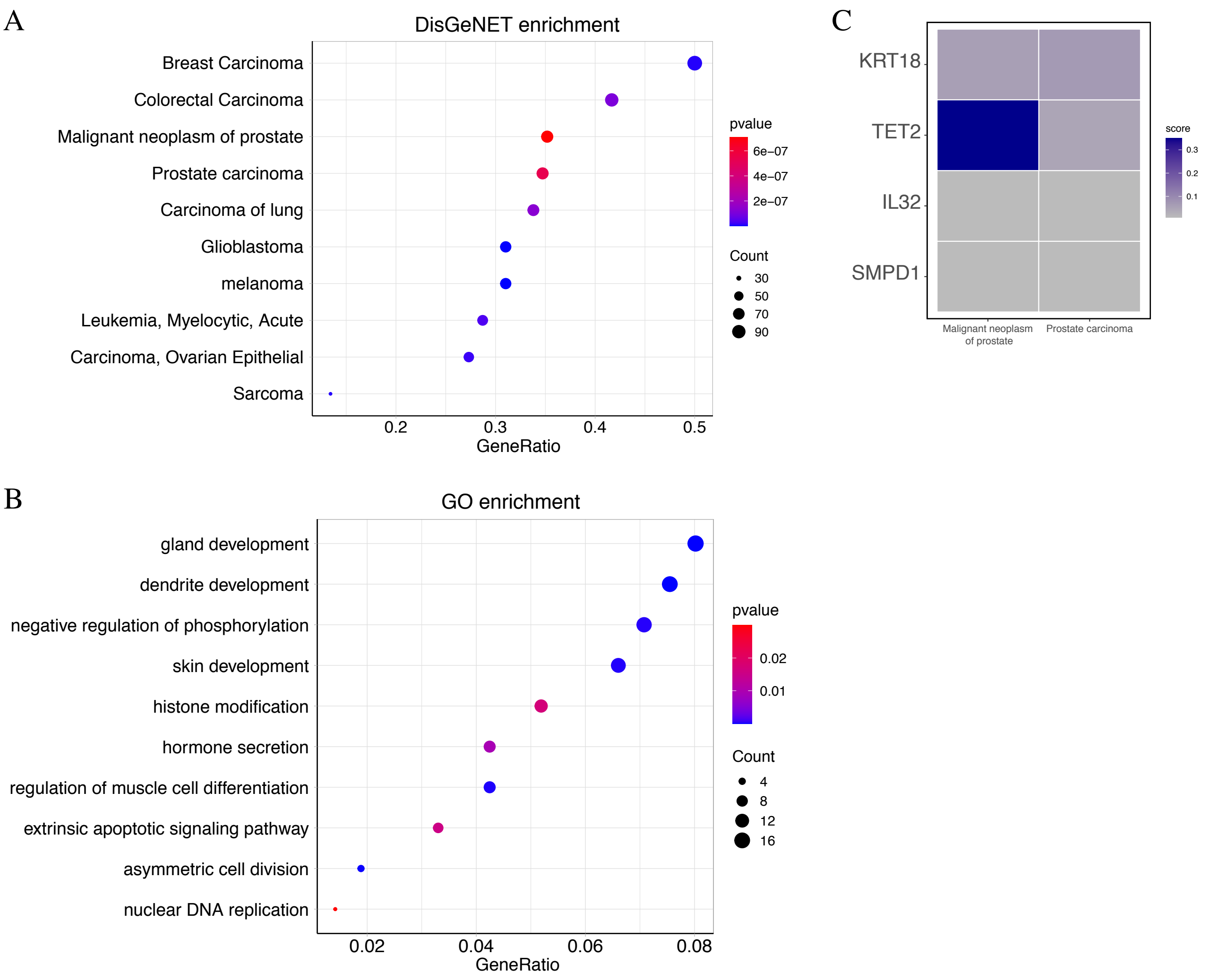

Supplementary Figure 2: Low risk variants analysis. (**A**) Top 10 neoplastic disease enrichment for the list of genes in the low-risk category. Count is the number of genes enriched for the specific disease. pvalue from multiple Fisher tests corrected for false discovery rate using the Benjamini-Hochberg method. (**B**) Gene ontology enrichment analysis of the genes with OR > 1.1, MMAF > 0.01 and CADD >10. (**C**) Gene-disease association heatmap for the list of genes in low-risk variants. Score based on the gene disease association performed with disgenet2r package.

Supplementary Table 1: evidence associated with high-risk genes and malignant neoplasm of prostate

Gene symbol	Disease name	Association type	PMID	Year	Score
ETV4	Malignant neoplasm of prostate	Biomarker	23814484	2013	0.4
ETV4	Malignant neoplasm of prostate	Biomarker	23244085	2012	0.4
ETV4	Malignant neoplasm of prostate	Altered Expression	25203299	2014	0.4
ETV4	Malignant neoplasm of prostate	Biomarker	16951139	2006	0.4
ETV4	Malignant neoplasm of prostate	Biomarker	17671502	2007	0.4
ETV4	Malignant neoplasm of prostate	Altered Expression	22895549	2012	0.4
ETV4	Malignant neoplasm of prostate	Biomarker	27783944	2016	0.4
ETV4	Malignant neoplasm of prostate	Biomarker	20736744	2010	0.4
ETV4	Malignant neoplasm of prostate	Biomarker	18794152	2008	0.4
ETV4	Malignant neoplasm of prostate	Altered Expression	21572435	2011	0.4
ETV4	Malignant neoplasm of prostate	Altered Expression	16585160	2006	0.4
ETV4	Malignant neoplasm of prostate	Biomarker	25595908	2015	0.4
ETV4	Malignant neoplasm of prostate	Altered Expression	25544710	2015	0.4
ETV4	Malignant neoplasm of prostate	Biomarker	28728983	2017	0.4
ETV4	Malignant neoplasm of prostate	Biomarker	17173048	2007	0.4
ETV4	Malignant neoplasm of prostate	Altered Expression	31016435	2019	0.4
ETV4	Malignant neoplasm of prostate	Biomarker	23918374	2013	0.4
ETV4	Malignant neoplasm of prostate	Biomarker	18483239	2008	0.4
ETV4	Malignant neoplasm of prostate	Biomarker	17108102	2006	0.4
ETV4	Malignant neoplasm of prostate	Altered Expression	31584209	2020	0.4
ETV4	Malignant neoplasm of prostate	Genetic Variation	22425584	2012	0.4
ETV4	Malignant neoplasm of prostate	Biomarker	18451133	2008	0.4
ETV4	Malignant neoplasm of prostate	Altered Expression	18711181	2008	0.4
ETV4	Malignant neoplasm of prostate	Biomarker	23578236	2013	0.4
ETV4	Malignant neoplasm of prostate	Genetic Variation	17334343	2007	0.4
ETV4	Malignant neoplasm of prostate	Biomarker	18172298	2008	0.4
NCOA2	Malignant neoplasm of prostate	Altered Expression	15663989	2004	0.38
NCOA2	Malignant neoplasm of prostate	Biomarker	19240160	2009	0.38
NCOA2	Malignant neoplasm of prostate	Altered Expression	17079484	2006	0.38
NCOA2	Malignant neoplasm of prostate	Biomarker	12237244	2002	0.38
NCOA2	Malignant neoplasm of prostate	Biomarker	30109944	2019	0.38
NCOA2	Malignant neoplasm of prostate	Biomarker	26799514	2016	0.38
NCOA2	Malignant neoplasm of prostate	Altered Expression	16598769	2006	0.38
NCOA2	Malignant neoplasm of prostate	Biomarker	20579941	2010	0.38
NCOA2	Malignant neoplasm of prostate	Altered Expression	12237244	2002	0.38
PPARA	Malignant neoplasm of prostate	Altered Expression	10955810	2000	0.36
PPARA	Malignant neoplasm of prostate	Altered Expression	31769890	2020	0.36
PPARA	Malignant neoplasm of prostate	Altered Expression	28483457	2017	0.36
PPARA	Malignant neoplasm of prostate	Biomarker	12897377	2003	0.36
PPARA	Malignant neoplasm of prostate	Biomarker	20466759	2010	0.36
PPARA	Malignant neoplasm of prostate	Biomarker	22919386	2012	0.36
PPARA	Malignant neoplasm of prostate	Biomarker	28072703	2017	0.36
CYP7B1	Malignant neoplasm of prostate	Biomarker	17639508	2007	0.32
CYP7B1	Malignant neoplasm of prostate	Altered Expression	17639508	2007	0.32
CYP7B1	Malignant neoplasm of prostate	Genetic Variation	15007371	2004	0.32

Supplementary Table 3: evidence associated with low-risk genes and malignant neoplasm of prostate

**Gene symbol**	**Disease name**	**Association type**	**PMID**	**Year**	**Score**
TET2	Malignant neoplasm of prostate	Genetic Variation	24832084	2014	0.35
TET2	Malignant neoplasm of prostate	Genetic Variation	27819678	2017	0.35
TET2	Malignant neoplasm of prostate	Genetic Variation	27486019	2016	0.35
TET2	Malignant neoplasm of prostate	Biomarker	30917865	2019	0.35
TET2	Malignant neoplasm of prostate	Biomarker	26404510	2015	0.35
TET2	Malignant neoplasm of prostate	Biomarker	23593118	2013	0.35
SMPD1	Malignant neoplasm of prostate	Genetic Variation	28872752	2018	0.01
KRT18	Malignant neoplasm of prostate	Biomarker	10928161	2000	0.06
KRT18	Malignant neoplasm of prostate	Biomarker	28494073	2017	0.06
KRT18	Malignant neoplasm of prostate	Biomarker	25857301	2015	0.06
KRT18	Malignant neoplasm of prostate	Altered Expression	26892177	2016	0.06
KRT18	Malignant neoplasm of prostate	Altered Expression	24243687	2014	0.06
KRT18	Malignant neoplasm of prostate	Biomarker	29848555	2018	0.06
IL32	Malignant neoplasm of prostate	Biomarker	20737563	2010	0.01
ANPEP	Malignant neoplasm of prostate	Altered Expression	23322201	2013	0.01
